# COVID-19 acts like a stress test, uncovering the vulnerable part of the human body: a retrospective study of 1640 cases in China

**DOI:** 10.1093/eurpub/ckae056

**Published:** 2024-04-12

**Authors:** Tian-Yi He, Hong-Yu Zhou, Ming-Hui Zhu, Ji-Li Zhang

**Affiliations:** Health Science Center, Ningbo University, Ningbo, China; The First Affiliated Hospital of Ningbo University, Ningbo, China; The First Affiliated Hospital of Ningbo University, Ningbo, China; Health Science Center, Ningbo University, Ningbo, China

## Abstract

**Background:**

Since the severe acute respiratory syndrome coronavirus 2 (SARS-CoV-2) infection exhibits multi-organ damage with diverse complications, the correlation between age, gender, medical history and clinical manifestations of novel coronavirus disease 2019 (COVID-19) patients was investigated.

**Methods:**

1640 patients who were infected with SARS-CoV-2 and hospitalized at the First Affiliated Hospital of Ningbo University from 22 December 2022 to 1 March 2023 were categorized and analysed. Normal distribution test and variance homogeneity test were performed. Based on the test results, one-way analysis of variance, Pearson's chi-squared test and logistic regression analysis were conducted in the study.

**Results:**

According to the ANOVA, there was a significant difference in the age distribution (*P* = .001) between different clinical presentations, while gender did not (*P* = .06). And regression analysis showed that age, hypertension, atherosclerosis and cancer were significant hazard factors for the development of predominant clinical manifestations in patients hospitalized with novel COVID-19. Additionally, infection with SARS-CoV-2 has the potential to exacerbate the burden on specific diseased or related organs.

**Conclusion:**

The elderly who are infected with SARS-CoV-2 ought to be treated with emphasis not only on antiviral therapy but also on individualized treatment that takes their medical history and comorbidities into account.

## Introduction

The novel coronavirus disease 2019 (COVID-19) has been referred to as the second pandemic of the 21st century by the WHO and has so far resulted in the infection of over 700 million people worldwide.^1^ Severe acute respiratory syndrome coronavirus 2 (SARS-CoV-2) is usually transmitted between humans horizontally via respiratory droplets or contact with contaminants.^2^ When the virus enters the body by binding to the angiotensin-converting enzyme-2 (ACE-2) receptors, mainly on type II alveolar epithelial cells, it triggers cellular and humoral-mediated immune responses.^3^ With the prolonged course of the disease, SARS-CoV-2 further destroys the alveolar wall, causing acute and severe pneumonia and even respiratory failure.^4^

The risk factors associated with COVID-19 require substantial consideration, not only for the prevention of infectious diseases but also for the clinical precision of treatment. It is suggested that age itself is a significant risk factor for poor outcomes in COVID-19 patients due to compromised immunity and physiologic reserve.^5^ Statistics published by the US Center for Disease Control and Prevention (CDC) also demonstrated that older patients (≥65 years) with COVID-19 have markedly higher intensive care unit admissions and mortality rates than younger patients.^5^ Research on gender differences suggested that sex hormones, gender-based lifestyles and environmental factors may contribute to greater severity and higher mortality in male patients, whereas women were more likely to be re-infected or to develop long-term COVID-19.^6^ Additionally, the fatality rates of COVID-19 patients with a history of diabetes, chronic respiratory and cardiovascular diseases, obesity, liver diseases, malignancy, human immunodeficiency viruses and renal diseases were reviewed in some research,^7^ but limited resources could be found for describing the statistical relationship between COVID-19 and comorbidities. Therefore, the correlation between the above factors needs to be standardized by statistical means.

As new research suggests that COVID-19 is an endothelial cell disease,^8^ the multi-organ damage it causes has also caught the public's attention. Studies on the aspect of cellular pathology, such as inflammation, oxidative stress, coagulation abnormalities, and cytokine storms, have been well-detailed.^8^ However, there is a lack of systematic clinical studies on the multiple clinical manifestations it causes. Therefore, herein we investigated the relationship between age, gender, comorbidities, medical history and clinical manifestations in a hospitalized population infected with SARS-CoV-2, thus providing a reference for COVID-19 prevention and treatment measures to achieve better disease stratification and improved outcomes.

## Methods

### Study design and patients

In this single-centre, retrospective study, patients infected with SARS-CoV-2 hospitalized at the First Affiliated Hospital of Ningbo University from 22 December 2022 to 1 March 2023 were enrolled. One patient hospitalized for malnutrition and 12 cases of death were excluded. Finally, 1640 patients were included and categorized into 13 groups by different main clinical manifestations, after which the differences in age, gender, and past medical history between the 13 groups were analysed.

The study was approved by the Ethics Committee of the First Affiliated Hospital of Ningbo University (approval number: 2023-152RS-01). All research procedures were performed in accordance with the criteria of the Declaration of Helsinki.

### Data collection

Information about the patients' age, gender, chief complaint, medical history, length of hospitalization, clinical outcomes and other relevant information were obtained from their electronic medical records. Patients with COVID-19 were diagnosed according to the *Diagnosis and Treatment Protocol for COVID-19* (9th edition).^9^ Throat swab samples were collected from the upper respiratory tracts of all the patients, following which pathogen detection using reverse transcription polymerase chain reaction (RT-PCR) was performed at the Ningbo Center for Disease Prevention and Control.

### Statistical analysis

Categorical variables were described by frequencies and percentages, while continuous variables were characterized by means and standard deviations (SD). All samples of continuous variables, including age and length of hospitalization, conformed to the normal distribution and homogeneity of variance. Thus, the variability of age and length of hospitalization of various groups was analysed by one-way analysis of variance (ANOVA). Differences in means between groups were compared two by two using LSD-t test. The means were categorized, with the same letter labelled for no significant statistical difference, and different letters labelled for significant statistical differences, in order, a, b and c from largest to smallest. Categorical variables, such as gender, were analysed using Pearson's chi-squared test for R*C contingency table, and finally Bonferroni method was performed for rate comparisons of multiple groups, which were sequentially marked as a and b.

In the multivariate analysis, age, gender, history of hypertension, diabetes, atherosclerosis, chronic lung disease and cancer were quantified in each population. Age was assigned as numeric, and gender as well as whether diseased or not was assigned values. After confirming that there is no multicollinearity between the independent variables, logistic regression was performed to calculate the odds ratio (OR) and *P* values in each group. All statistical analyses were performed using Microsoft Excel 2019, SPSS software (version 22.0; IBM, Armonk, NY), and GraphPad Prism 9.5.1 (GraphPad Software, CA, USA). *P* values <.05 was considered statistically significant.

## Results

### Demographic data and the research objects

**Figure 1:**
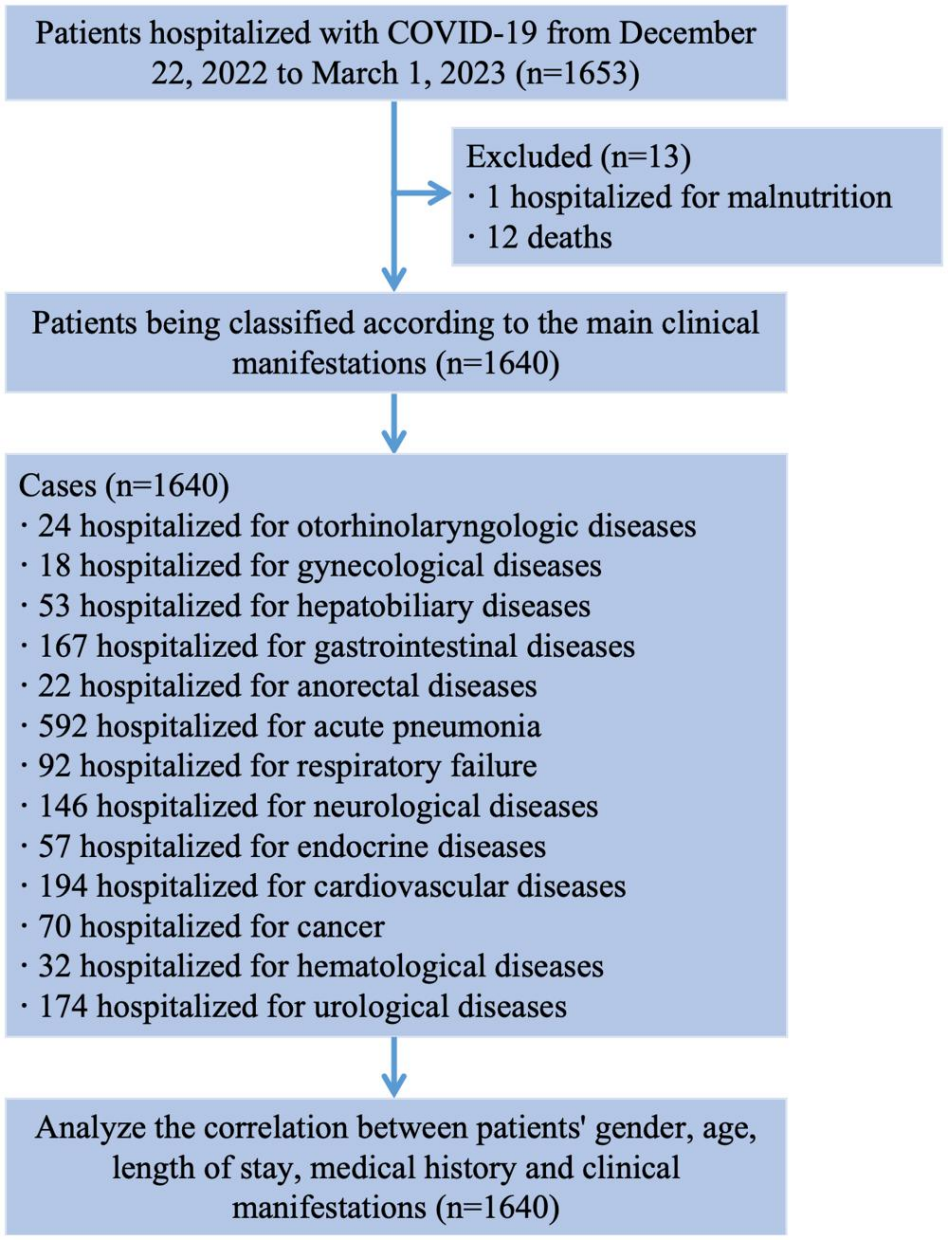
The flow diagram of the study.

Of 1653 patients, 13 were excluded, and the remaining 1640 patients were analysed (figure 1). The mean age of these patients was 67.7 (SD = 22.3) years, ranging from 1 to 105 years old. There were 594 (36.22%) patients aged 75–89 years old, followed by 523 (31.89%) patients aged 60-74 years old, 180 (10.98%) patients aged 45–59 years old, 152 (9.27%) patients aged ≥90 years old, 119 (7.26%) patients aged <14 years old, and 72 (4.39%) patients aged 15–44 years old. Among these patients, 934 (56.95%) were male. The most prevalent comorbidity was hypertension (*n* = 855, 52.13%), and other comorbidities such as diabetes, atherosclerosis, chronic pulmonary disease, and atherosclerosis also occupied more than 20% of the hospitalized patients. 354 (21.59%) patients had a combination of three or more comorbidities, suggesting that these patients hospitalized for SARS-CoV-2 infection exhibited overall poorer physical functioning than normal senior people. Also, the number and severity of these comorbidities are likely to have a strong positive correlation with COVID-19 progression.

### Comparison of data on age and gender

The average age, gender percentage, and length of hospitalization of patients with each clinical manifestation were counted and tabulated (table 1). There was a significant difference in age but not gender between each group, reflecting the age-specific nature of the patients' manifestations after infection with SARS-CoV-2.

**Table 1:** Comparison of age, gender, and length of hospitalization among different groups

	Age				Male gender		Hospital stay		
	Average	Median	SD	*P-*value	*N* (%)	*P*-value	Average	SD	*P*-value
Otorhinolaryngologic diseases (*n* = 24)	74.1	75.0	10.9	.001	15(62.5)	.06	7.5	3.4	<.001
Gynecological diseases (*n* = 18)	66.7	72.5	17.1		/		7.2	3.6	
Hepatobiliary diseases (*n* = 53)	70.8	73.0	11.8		34(64.2)		11.6	8.5	
Gastrointestinal diseases (*n* = 167)	72.2	73.0	13.2		89(53.3)		9.9	5.2	
Anorectal diseases (*n* = 22)	64.3	70.0	16.1		15(68.2)		7.9	6.0	
Acute pneumonia (*n* = 592)	59.2	69.0	30.3		355(60.0)		10.5	6.7	
Respiratory failure (*n* = 92)	78.7	80.0	11.8		52(56.5)		13.6	9.8	
Neurological diseases (*n* = 146)	74.0	75.0	12.7		69(47.3)		11.8	7.2	
Endocrine diseases (*n* = 57)	68.0	70.0	14.9		25(43.9)		9.7	5.5	
Cardiovascular diseases (*n* = 194)	77.4	78.0	12.6		119(61.3)		9.5	5.9	
Cancer (*n* = 70)	69.5	70.0	10.2		41(58.6)		8.8	5.7	
Hematological diseases (*n* = 32)	61.3	57.0	18.5		15(46.9)		8.0	4.8	
Urological diseases (*n* = 174)	69.3	71.0	15.1		106(60.9)		9.8	5.5	

Infected individuals with respiratory failure, cardiovascular disease, otorhinolaryngologic disease, and neurologic disease were significantly older, with average ages of 78.7, 77.4, 74.1 and 74.0, respectively, while those with anorectal disease, hematology, and acute pneumonia were significantly younger, with average ages of 64.3, 61.3 and 59.2, respectively (Supplementary figure 1). This may indicate that as people age, the vulnerability and sensitivity of the heart, brain and blood vessels increase markedly compared to other parts of the body,^10–12^ to the point where they manifest severe disease following viral incursions. The young age of patients with acute pneumonia and hematological disease was mainly due to the high incidence of neonatal pneumonia and hereditary lymphoma.

**Figure 2:**
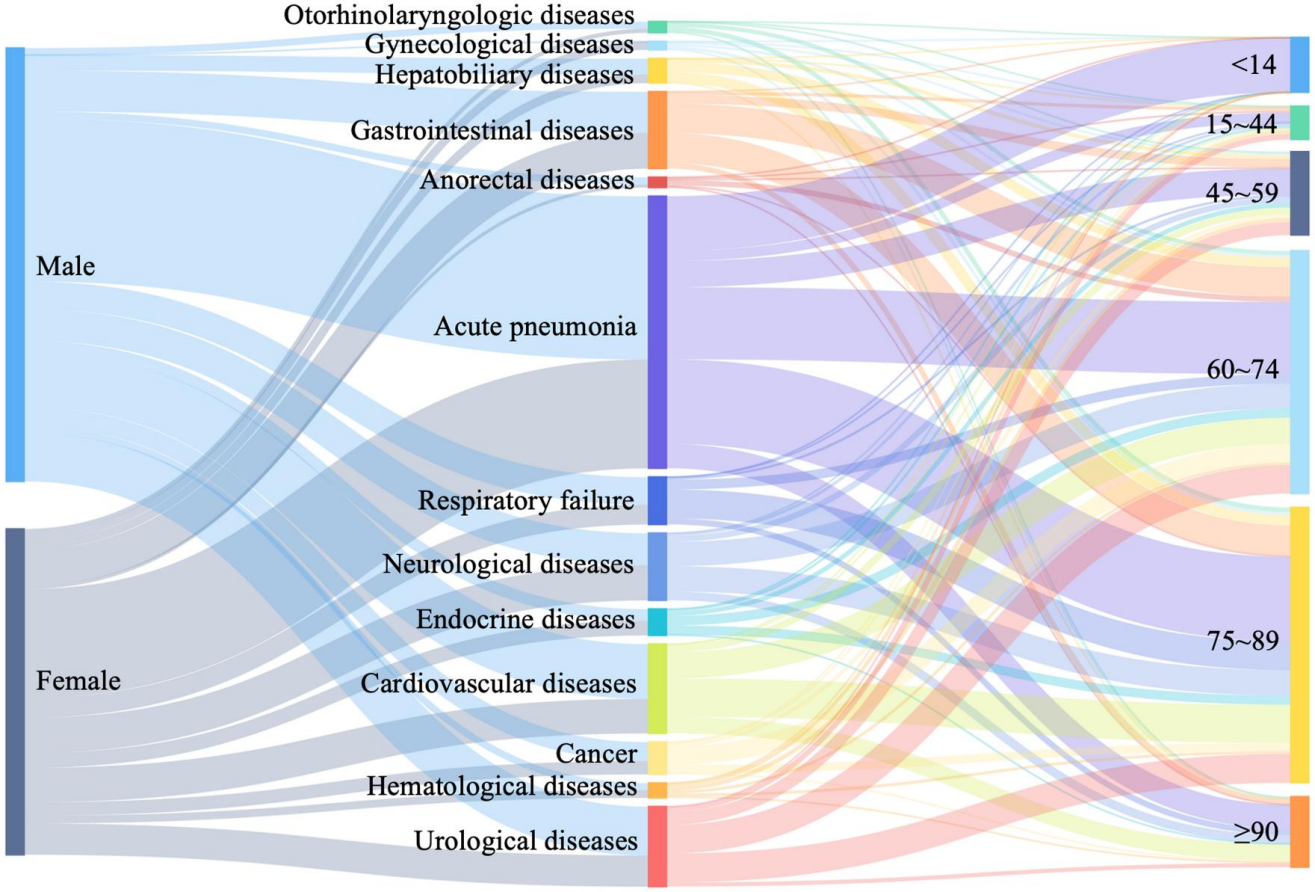
Sankey diagram of gender-clinical manifestations-age relationships.

After excluding gynecological disease, there was no significant difference in the gender distribution of patients exhibiting different clinical manifestations (Supplementary figure 2). The highest proportion of women, reaching 56.1%, was among the patients who exhibited endocrine disorders. This may be related to the large fluctuations in hormone levels that women experience during infancy, puberty, reproductive periods and menopause. Also, the intake of estrogens and progesterone as therapeutic agents may increase women's susceptibility to endocrine disorders during the period of COVID-19.^13^^,^^14^ Subsequent to endocrine diseases, hematologic and neurological diseases also involved a high proportion of female patients, accounting for 53.1% and 52.7%, respectively. Integrating the relationship between age, gender, and clinical manifestations, the macroscopic distribution of data was presented in the form of a Sankey diagram (figure 2).

### Multivariate logistic regression analysis of risk factors

In addition to age and gender, the various comorbidities also increase the risk for infected patients,^7^ so it is essential to ascertain the true independent factors associated with clinical manifestations. The variables were assigned (Supplementary table 1), and multifactorial logistic regression analysis was conducted (Supplementary table 2). Age, hypertension, atherosclerosis, and cancer were found to be independent hazard factors for various clinical manifestations. The manifestations of gastrointestinal, respiratory, neurological and cardiovascular diseases were associated with age with OR of 1.016, 1.047, 1.019 and 1.030, respectively. The history of hypertension was associated with manifestations of cardiovascular disease and urological disease with OR 1.457 and 1.797. Also, diabetes was a significant independent risk factor for endocrine diseases (OR = 12.828), and chronic pulmonary disease was a significant independent risk factor for acute pneumonia (OR = 1.982). In addition, history of atherosclerosis correlates with neurological (OR = 1.651), cardiovascular (OR = 1.441), and urological diseases (OR = 1.425).

Age was analysed as an independent factor for respiratory failure, cardiovascular disease, and neurological disorders, which is consistent with the results of the previous univariate analyses, emphasizing that ageing increases susceptibility of these organs. Additionally, increasing age appears to be an independent factor in the manifestation of gastrointestinal disorders in COVID-19 patients, which is consistent with studies of changes in gastrointestinal glandular secretion with ageing.^15^

Surprisingly, femininity is not an independent factor for endocrine diseases but rather for neurological diseases. Studies have mentioned that the brain structures of men and women develop differently from the embryonic period.^16^ However, social roles, such as healthcare workers, as well as life events after entering society, such as pregnancy and childbirth, also stimulate different responses in women from men when faced with stresses.^17–19^ It may serve as a more important role and result in a discrepancy of gender factors in neuropathology among COVID-19 patients.

The relationship between comorbidities and clinical manifestations is mainly reflected in diseases with associated physiologic functions and pathologic alterations. Examples include exacerbation of kidney and heart diseases due to elevated blood pressure and narrowed blood vessel lumens, disruption of endocrinology by metabolic disorders, worsening of acute lung inflammation by chronic lung lesions, and facilitation of neoplastic development by pre-existing malignancy or immunodeficiency. Among them, COVID-19 acts as an indispensable stimulation in inducing the exacerbation of previous lesions or even affecting other related systems.

### The length of hospitalization and medical history of COVID-19 clinical manifestations

Drawn by the correlation between localized lesions and clinical symptoms in the results of multifactorial regression analyses, the factors influencing the progression of clinical manifestations in COVID-19 patients were further investigated. The distribution of length of hospitalization for patients with different major clinical manifestations was presented in the violin plot (Supplementary figure 3), which hinted at variances in disease severity between the categories. Patients with respiratory failure had a significantly higher length of hospitalization, averaging 13.6 days. They were closely followed by neurological disorders, hepatobiliary disorders and acute pneumonia, with 11.8, 11.6 and 10.5 days, respectively. This was largely due to the complexity of complications and past history of these patients. For instance, patients with respiratory failure were more likely to develop septic shock, acidosis, acute heart failure, and even multi-organ failure than others, which forced them to prolong their hospitalization.

**Figure 3:**
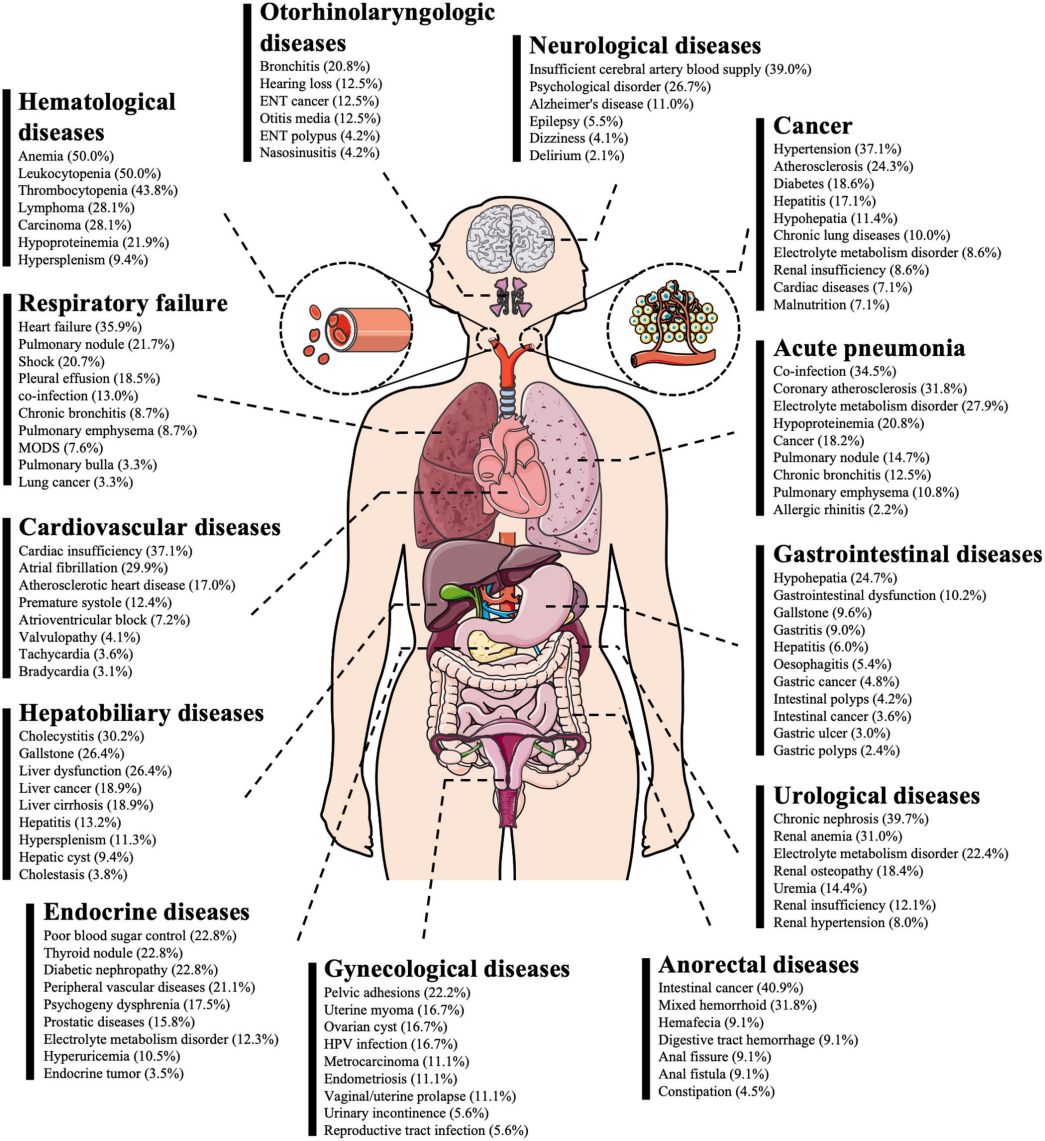
The profile of the past medical history of patients with different clinical manifestations.

The past medical histories of patients presenting various clinical manifestations were fully accounted for (figure 3), indicating that majority of the hospitalized patients had underlying conditions that tend to induce similar manifestations after infection. For instance, 39.0% of patients who developed neurological disorders had a history of cerebral arterial insufficiency and 26.7% had a history of psychiatric disorders. 35.9% of patients who developed respiratory failure had previous heart failure, 21.7% had pulmonary nodules and 20.7% had shock. Inadequate organ function, local neoplasms, local vascular lesions, disturbances in homeostasis, co-infections, chronic inflammation, polyps and cysts may all be quantitative changes that cause aggravation of COVID-19 symptoms. It is also noteworthy that previously localized lesions can be mutually responsible and reinforce newly progressed symptoms, creating an additive and cumulative effect.

## Discussion

As the largest global public health outbreak in the 21st century, the COVID-19 epidemic has disrupted human life and social development.^4^ Based on the fact that COVID-19 is an epithelial cell disease and can lead to multi-organ damage,^8^ the relationship between multiple potential risk factors and different clinical manifestations was investigated. Demographic data show the positive correlation between the severity of comorbidities and COVID-19 progression, which may result from the increased ACE-2 receptors expressed on certain organs caused by corresponding diseases, such as hypertension, COPD, diabetes, liver diseases, and renal diseases, thereby making the individuals more susceptible to infection.^7^ By univariate analysis, this study roughly concluded that the presence of different clinical manifestations in COVID-19 patients was age-related, whereas the gender factor did not differ among manifestations but was more prevalent in women with endocrine diseases.

Subsequently, the interference of confounding factors was excluded in the results of multifactorial logistic regression analysis and whether common comorbidities were independent factors stimulating the divergence of clinical manifestations after infection was investigated. The univariate and multivariate analyses were consistent in their findings that ageing was a contributor to the development of respiratory failure, cardiovascular disease, and neurological disease. Also, multifactorial analysis showed that gender had no significant effect on the clinical manifestations in patients. Notably, a history of diabetes had a significant hazardous effect with an OR of 12.838 for patients exhibiting endocrine disorders. The mechanism may be related to the role of diabetes in promoting inflammation and immune insufficiency as well as reducing the clearance of viruses.^20^ In addition, acute pneumonia was highly correlated with chronic lung disease and not with age, contrary to the results for respiratory failure. It may imply that chronic lung lesions are a hazard factor for acute lung pneumonia and that the development from acute pneumonia to respiratory failure or even death is more dependent on the deterioration of physical function and decreased immunity with ageing.^5^^,^^21^^,^^22^ The occurrence of neurological disorders is related to gender, probably more due to the fact that women play social roles and confront social problems differently from men, thus being more prone to anxiety, panic, or other emotions, and to a lesser extent due to the differences in the physiological structure of the brain between men and women.^17–19^ These neuropsychological stimuli may intensify their neuropathy when they are confronted with COVID-19.

Finally, attracted by the results of the multifactorial analysis, the relationship between complications, medical history, and clinical manifestations was further explored. The results of the violin plot suggest that the length of hospitalization broadly correlates with the severity of the systemic lesions and complications. But some patients may have been hospitalized for reasons not directly related to COVID-19, such as trauma, and experienced surgery, thus experiencing longer lengths of hospitalization. However, this does not affect the overall results. Based on the fact that SARS-CoV-2 infection causes multiple symptoms as well as the statistical relationship between the patients' past medical history and their current clinical manifestations in figure 3 and Supplementary table 2, it can be crudely hypothesized that COVID-19 acted as a potential factor inducing the underlying disease or exacerbating the pre-existing disease.

However, the current study still has limitations: (i) the sample size was limited; (ii) there was a selection bias in the sample by not including healthy people as well as asymptomatic infected people in the study; (iii) the purpose of the patients coming to the hospital are varied, part of them may be hospitalized for diseases directly caused by other factors, with COVID-19 playing only a potential or secondary role (iv) some hospitalized patients experienced surgery. For these patients, the size of the surgery, postoperative complications, and the physiological condition all affected the length of hospitalization. The findings do not allow for an accurate ranking of the severity of the systems damaged by the virus. Therefore, exact mechanisms need to be demonstrated through further researches and (v) since this research is a single-centre clinical study, further evidence from multi-centre clinical trials is needed. A series of prognostic evaluations and long-term follow-up are needed to assess the effects of COVID-19 more accurately after patients are discharged from the hospital. The innovativeness of this study lies in the systematic description of the distribution of patients with different clinical manifestations and the statistical correlation between multiple factors and these clinical manifestations. Besides, it illustrates COVID-19 as a multi-organ stress test that manifests itself specifically in the vulnerable parts of the body at the clinical aspect. Therefore, when an outbreak arrives, it is not enough to emphasize the protection of the elderly or immunocompromized groups, as individuals will manifest idiosyncratic symptoms when the virus breaks through the body's defenses. This is the reason why it is essential to both adopt uniform antiviral treatment and emphasize individualized and precision medicine. Hopefully, this article will provide a reference for the prevention and control of COVID-19 or other influenza cases that are seasonally circulating or disseminated in the post-pandemic era.

The results of the study showed that age-related variability between different COVID-19 clinical manifestations may be due to increased susceptibility of cardiovascular endothelial cells, gastrointestinal glands, and brain neurons with ageing. Also, the tendency of the infected women to exhibit neurological lesions may be due to their social roles and life events. Furthermore, multifactorial regression analysis revealed the role of COVID-19 in exacerbating pre-existing disorders. Finally, we calculated and summarized the medical history of patients with different clinical manifestations.

## Supplementary Material

ckae056_Supplementary_Data

## Data Availability

The data underlying this article were provided by the First Affiliated Hospital of Ningbo University under licence. Data will be shared on request to the corresponding author with permission of the First Affiliated Hospital of Ningbo University. Key points COVID-19 aggravates organ dysfunction caused by aging, mainly reflected in cardiovascular diseases, nervous system diseases and gastrointestinal diseases. Overall, the clinical manifestations of COVID-19 do not differ significantly between the genders, but women's role in social life may make them more susceptible to neuropathy after infection with the virus. COVID-19 plays a role like a stress test in the development of its clinical manifestations. For different individuals, COVID-19 is more likely to show symptoms first in a person's less functioning or more vulnerable organs. In the post-pandemic era, individualized treatment is an effective way to treat the epidemic of COVID-19.
