# Supplementary material for: COVID-19 acts like a stress test, uncovering the vulnerable part of the human body: a retrospective study of 1640 cases in China
Source: Eur J Public Health. 2024 Apr 12;34(4):760–5. doi: 10.1093/eurpub/ckae056 (PMC11293811; doi:10.1093/eurpub/ckae056)
Supplement: ckae056_Supplementary_Data [file ckae056_supplementary_data.zip › ckae056_Supplementary_Data/ejph-2023-11-om-0622-File009.pdf]

Supplementary Table 1. Variables and assignment settings for hazard factor analysis

**Variables**	**Description**
Independent variables	
Age	Numerical
Gender	0=Female, 1=Male
Hypertension	0=No, 1=Yes
Diabetes	0=No, 1=Yes
Atherosclerosis	0=No, 1=Yes
Chronic pulmonary diseases	0=No, 1=Yes
Cancer	0=No, 1=Yes
Dependent variables	
Otorhinolaryngologic diseases	0=No, 1=Yes
Gynecological diseases	0=No, 1=Yes
Hepatobiliary diseases	0=No, 1=Yes
Gastrointestinal diseases	0=No, 1=Yes
Anorectal diseases	0=No, 1=Yes
Acute pneumonia	0=No, 1=Yes
Respiratory failure	0=No, 1=Yes
Neurological diseases	0=No, 1=Yes
Endocrine diseases	0=No, 1=Yes
Cardiovascular diseases	0=No, 1=Yes
Cancer	0=No, 1=Yes
Hematological diseases	0=No, 1=Yes
Urological diseases	0=No, 1=Yes
