# Supplementary material for: COVID-19 acts like a stress test, uncovering the vulnerable part of the human body: a retrospective study of 1640 cases in China
Source: Eur J Public Health. 2024 Apr 12;34(4):760–5. doi: 10.1093/eurpub/ckae056 (PMC11293811; doi:10.1093/eurpub/ckae056)
Supplement: ckae056_Supplementary_Data [file ckae056_supplementary_data.zip › ckae056_Supplementary_Data/ejph-2023-11-om-0622-File010.pdf]

Supplementary Table 2. OR from multifactorial logistic regression analysis of the main clinical manifestations

	**Age**	**Gender**	**Hypertension**	**Diabetes**	**Atherosclerosis**	**Chronic pulmonary diseases**	**Cancer**
Otorhinolaryngologic diseases	1.013 (*P*=0.383)	1.137 (*P*=0.772)	2.697 (*P*=0.054)	0.972 (*P*=0.951)	0.232 (*P*=0.020)	1.353 (*P*=0.543)	1.406 (*P*=0.466)
Gynecological diseases	1.003 (*P*=0.810)	0 (*P*=0.989)	2.030 (*P*=0.204)	0.692 (*P*=0.537)	0.276 (*P*=0.095)	0 (*P*=0.993)	1.256 (*P*=0.726)
Hepatobiliary diseases	1.016 (*P*=0.054)	1.427 (*P*=0.236)	0.617 (*P*=0.116)	1.531 (*P*=0.772)	0.233 (*P*=0.001)	0.772 (*P*=0.506)	1.410 (*P*=0.281)
Gastrointestinal diseases	1.016 (*P*=0.001)	0.852 (*P*=0.345)	0.837 (*P*=0.321)	0.885 (*P*=0.528)	0.938 (*P*=0.724)	0.902 (*P*=0.636)	0.747 (*P*=0.184)
Anorectal diseases	1.000 (*P*=0.963)	1.675 (*P*=0.273)	0.955 (*P*=0.925)	0.486 (*P*=0.263)	0.242 (*P*=0.062)	0.407 (*P*=0.240)	2.735 (*P*=0.029)
Acute pneumonia	0.971 (*P*<0.001)	1.202 (*P*=0.102)	0.902 (*P*=0.390)	1.019 (*P*=0.886)	0.909 (*P*=0.442)	1.982 (*P*<0.001)	1.026 (*P*=0.848)
Respiratory failure	1.047 (*P*<0.001)	1.094 (*P*=0.690)	0.981 (*P*=0.936)	0.879 (*P*=0.601)	0.772 (*P*=0.277)	0.315 (*P*=0.002)	0.615 (*P*=0.110)
Neurological diseases	1.019 (*P*=0.001)	0.659 (*P*=0.021)	0.931 (*P*=0.709)	0.780 (*P*=0.228)	1.651 (*P*=0.007)	0.761 (*P*=0.255)	0.638 (*P*=0.071)
Endocrine diseases	0.993 (*P*=0.403)	0.689 (*P*=0.191)	0.588 (*P*=0.090)	12.838 (*P*<0.001)	0.798 (*P*=0.471)	0.602 (*P*=0.270)	0.225 (*P*=0.014)
Cardiovascular diseases	1.030 (*P*<0.001)	1.081 (*P*=0.638)	1.457 (*P*=0.030)	0.679 (*P*=0.035)	1.441 (*P*=0.024)	1.302 (*P*=0.154)	0.935 (*P*=0.729)
Cancer	1.011 (*P*=0.138)	0.994 (*P*=0.980)	0.453 (*P*=0.005)	0.693 (*P*=0.263)	0.709 (*P*=0.264)	0.439 (*P*=0.039)	5.233 (*P*<0.001)
Hematological diseases	0.991 (*P*=0.279)	0.702 (*P*=0.334)	0.680 (*P*=0.353)	1.060 (*P*=0.895)	1.509 (*P*=0.316)	0.711 (*P*=0.545)	0.871 (*P*=0.783)
Urological diseases	0.997 (*P*=0.519)	1.215 (*P*=0.249)	1.797 (*P*=0.002)	1.038 (*P*=0.840)	1.425 (*P*=0.042)	0.698 (*P*=0.121)	0.085 (*P*<0.001)
