# Supplementary figures and images for: COVID-19 acts like a stress test, uncovering the vulnerable part of the human body: a retrospective study of 1640 cases in China
Source: Eur J Public Health. 2024 Apr 12;34(4):760–5. doi: 10.1093/eurpub/ckae056 (PMC11293811; doi:10.1093/eurpub/ckae056)

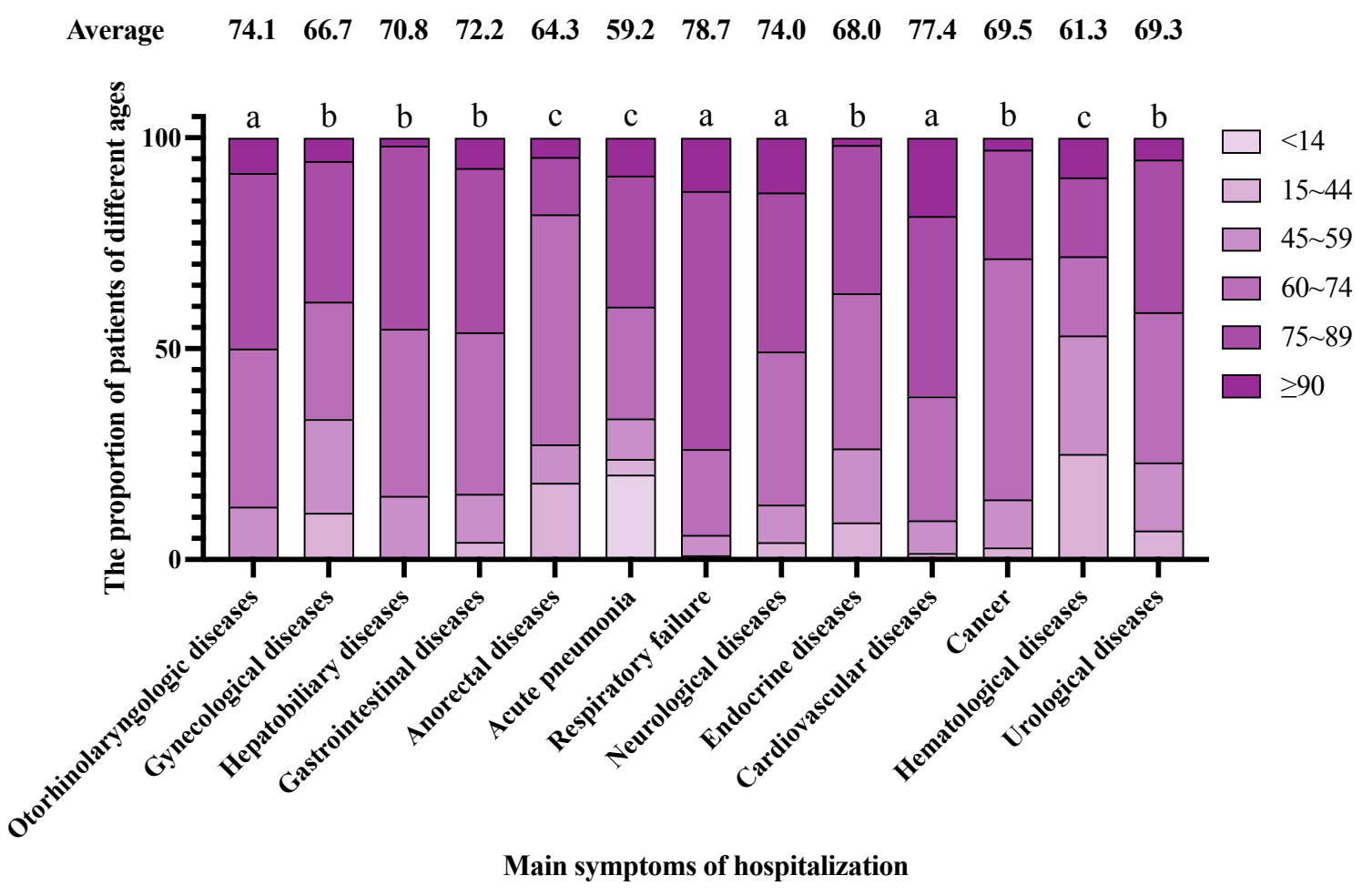

Supplement: ckae056_Supplementary_Data [file ckae056_supplementary_data.zip › ckae056_Supplementary_Data/ejph-2023-11-om-0622-File006.pdf]

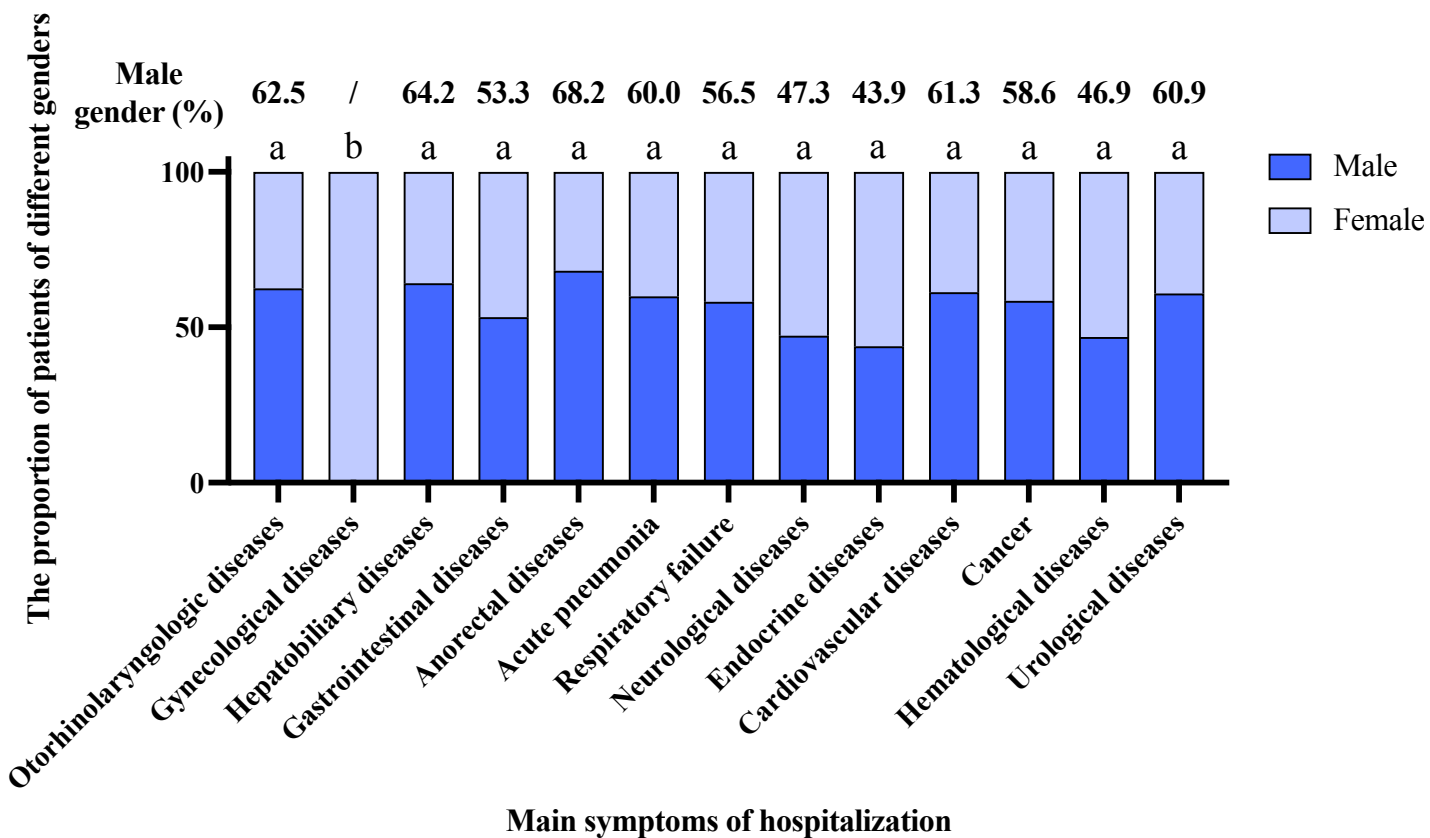

Supplement: ckae056_Supplementary_Data [file ckae056_supplementary_data.zip › ckae056_Supplementary_Data/ejph-2023-11-om-0622-File007.pdf]

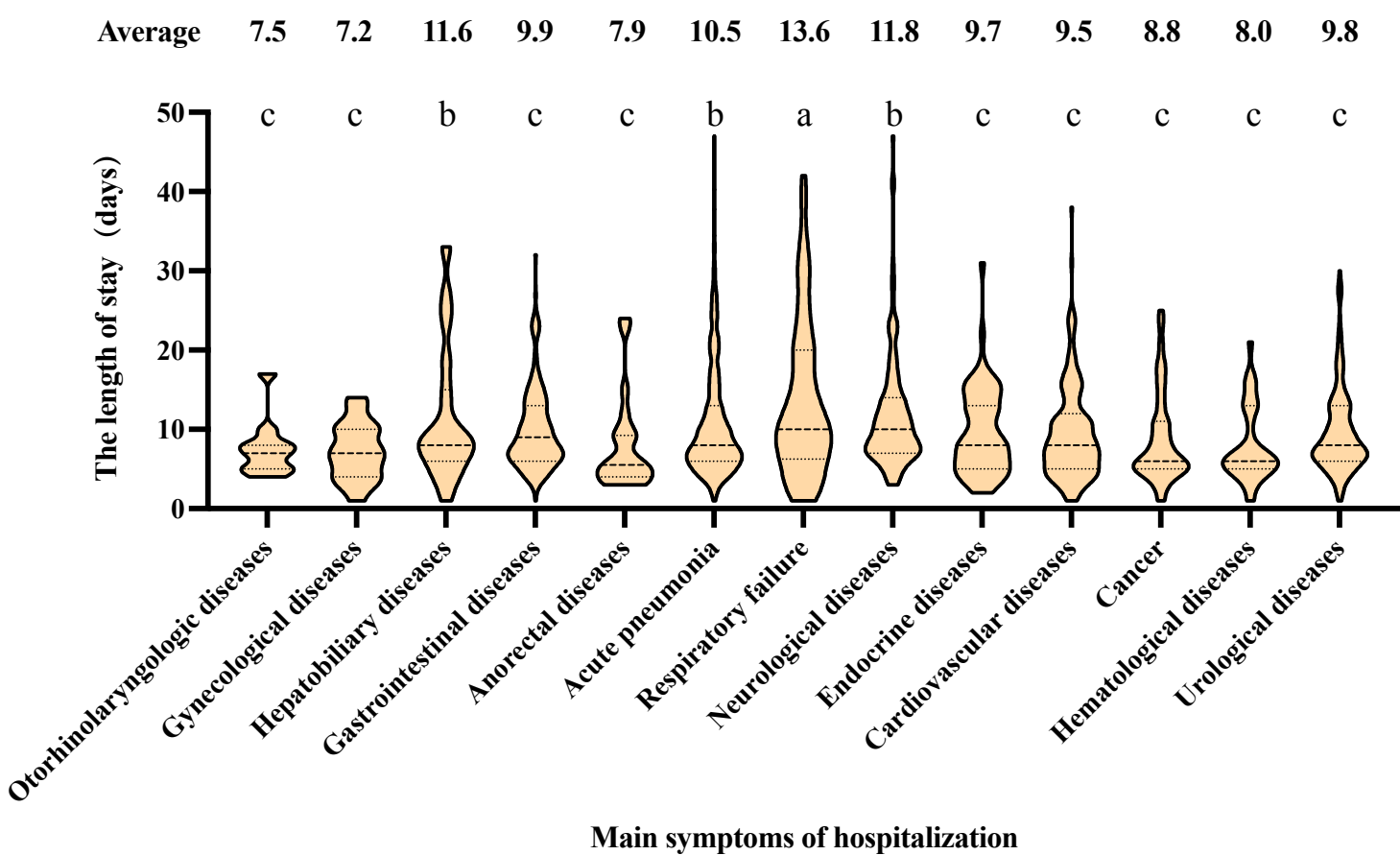

Supplement: ckae056_Supplementary_Data [file ckae056_supplementary_data.zip › ckae056_Supplementary_Data/ejph-2023-11-om-0622-File008.pdf]
